# Mutually exclusive driver mutations identifies 2 separate primaries in a collision tumor initially interpreted as a solitary lung adenocarcinoma with tumor heterogeneity

**DOI:** 10.1016/j.rmcr.2024.101986

**Published:** 2024-01-19

**Authors:** Mary M. Torrez, Khalil Sheibani, Mohammad A. Vasef

**Affiliations:** a Department of Pathology, University of New Mexico, Albuquerque, NM, USA; b Department of Pathology, Orange County Global Medical Center, Santa Ana, CA, USA

**Keywords:** EGFR mutation, KRAS mutation, Molecular profiles, Lung adenocarcinoma, Non–small cell lung cancer, Collision/synchronous tumors

## Abstract

Distinction of histologically heterogenous, single primary tumor from two or more collision tumors with different primaries could represent a challenge to practicing pathologists. Histologic variations including differences in degree of differentiating within a tumor, are typically interpreted as tumor heterogeneity in a contiguous small size tumor biopsy. The authors report a case of adult former smoker female who presented with lung mass and a metastatic lytic lesion of acetabulum. A needle biopsy of a lung mass revealed an adenocarcinoma with well and moderately differentiated components. Next generation sequencing studies proved 2 different primaries in this small needle biopsy.

## 1 Introduction

Lung cancer continues to be the leading cause of cancer death In the United States and world (1,2) with non-small cell lung cancer (NSCLC) accounting for majority of cases [1]. Lung adenocarcinoma can be morphologically heterogenous [3]. Variation in histomorphology and immunophenotype is typically attributed to tumor heterogeneity, particularly in a contiguous tumor. Most tumors with or without multiple primaries present as separate tumor nodules. However, rare cases may present as contiguous/collision tumors. Distinction of collision tumors from tumor heterogeneity can be difficult on morphologic and immunophenotypic grounds alone. Therefore, the differential diagnosis of heterogenous tumors includes tumor to tumor metastases from a solitary primary site as well as the rare category of synchronous and collision tumors with two or more primaries. Testing for targetable driver mutations in lung adenocarcinoma including EGFR and KRAS mutations is now standard of care as there are major diagnostic and therapeutic implications [4,5].

Herein, we report a 74-year-old female with pulmonary collision tumor consisting of a well-differentiated adenocarcinoma and moderately-differentiated adenocarcinoma confirmed by Next Generation Sequencing (NGS) with the former component showing two sensitizing EGFR mutations (G719C and S768I variants) and the latter showing a KRAS G12A mutation. We highlight the utility of molecular profiling on DNA obtained from separately micro- or macro-dissected foci of heterogenous appearing tumors to differentiate true tumor heterogeneity from synchronous/collision tumors.

## 2 Case presentation

The patient is a 74-year-old female former smoker with a past medical history of arthritis, gastroesophageal reflux, and hiatal hernia who presented to the emergency department with intractable vomiting, new onset diarrhea with bowel incontinence, weight loss, and shortness of breath. The patient has no significant family history. On exam, the patient was slightly hypoxic with an oxygen saturation of 88 %, requiring 2 L of oxygen. An extensive laboratory workup was negative; however, a portable chest x-ray revealed a right apical nodular opacity.

**Fig. 1:**
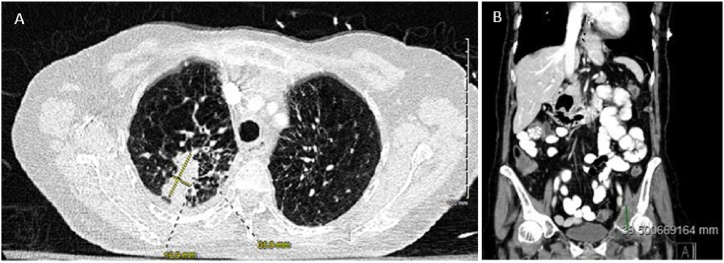
**A-B**: Computed tomography (CT) angiogram of the chest reveals two masses in the right lung apex measuring 2.3 × 2.1 cm and 3.6 × 1.7 cm **(A)**, and a CT of the abdomen and pelvis demonstrates a destructive lytic lesion of the left acetabulum measuring 3.9 × 3.4 cm **(B)**.

A follow-up computed tomography (CT) angiogram of the chest was remarkable for two masses in the right lung apex measuring 2.3 × 2.1 cm and 3.6 × 1.7 cm respectively (Fig. 1A). In addition, a CT of the abdomen and pelvis demonstrated a destructive lytic lesion of the left acetabulum measuring 3.9 × 3.4 cm (Fig. 1B) and a 1.3 cm cystic lesion in the uncinate process of the pancreas without a solid component.

**Fig. 2:**
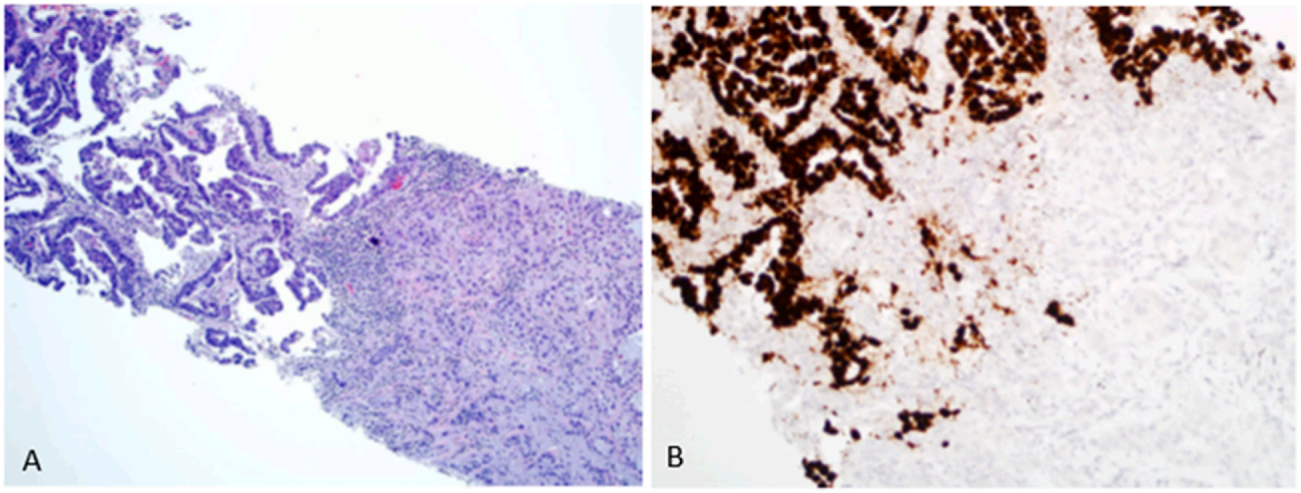
**A-B**: Histologic sections of the needle core biopsy of the right lung mass reveals two morphologically distinct areas including a well-differentiated adenocarcinoma in the left half of the image and a moderately-differentiated adenocarcinoma on the right half of the image **(A)**. Immunohistochemistry for TTF1 shows positivity in the well-differentiated adenocarcinoma component of the needle biopsy **(B)**.

Needle biopsies of left acetabulum lytic lesion and lung mass were performed. A biopsy of the left acetabulum showed metastatic moderately-differentiated adenocarcinoma. Paraffin immunohistochemical stains revealed the tumor to be positive for CK19 (Lecia Novocastra, Deer Park, IL USA), CK7 (Dako/Agilent, Santa Clara, CA, US), focal weak CK20 (Dako/Agilent, Santa Clara, CA, US), and weak SATB2 (Sigma, Rocklin, CA, USA), and negative for TTF1 (Leica Biosystems, Buffalo Grove, IL, USA), CDX2 (Ventana Medical Systems, Oro Valley, AZ, USA), PAX8 (Abcam, Cambridge, MA, USA), GATA3 (Ventana Medical Systems, Oro Valley, AZ, USA) and Napsin A (Lecia Novocastra, Deer Park, IL USA). In addition, immunohistochemical stains for mismatch repair proteins (MMRPs) including PMS2, MSH2, MSH6, and MLH1 (Ventana Medical Systems, Oro Valley, AZ, USA/BD Pharmingen, San Diego, CA, USA) showed intact and preserved MMRPs. One week later, the lung mass was biopsied and revealed adenocarcinoma with two distinct histologic patterns including a well-differentiated and a moderately-differentiated component (Fig. 2A). TTF1 and Napsin A were strongly positive in the well-differentiated component and negative in the moderately-differentiated component (Fig. 2B).

CK7, small subset CK20, CK19, and SATB2 were focally positive in both components, and CDX2, PAX8, and GATA3 were negative. Immunohistochemistry for PD-L1 (Ventana Medical Systems, Oro Valley, AZ, USA) revealed a tumor proportion score of 20–29 %. The histomorphologic features of the moderately-differentiated component of the lung biopsy were similar to the moderately-differentiated adenocarcinoma of the acetabulum.

### 2.1 Molecular Pathology

Molecular analysis by next generation sequencing (NGS) performed on genomic DNA isolated from formalin fixed paraffin embedded (FFPE) tumor sample using custom-designed AmpliSeq primer sets in the Ion Torrent NGS platform on the lung biopsy specimen identified three driver mutations including a c.2155G > T (G719C) mutation in exon 18 of the EGFR gene with a variant allele frequency (VAF) of 17 %, a c.2303G > T (S768I) mutation in exon 20 of the EGFR gene with a VAF of 20 %, and a c. 35G > C (p.G12A) mutation in codon 2 of KRAS with a VAF of 12 %. No mutations were detected in codons 582–620 (including codon 600) of exon 15 or codons 439–477 of exon 11 of the BRAF gene. Fluorescence In Situ Hybridization (FISH) analysis (performed on FFPE tumor tissue sections using FISH break-apart probes for ALK, ROS1, RET fusions and targeted probes for MET amplification (Vysis probes, Abbott Molecular Inc., Illinois, USA) revealed no evidence for rearrangements of ALK, ROS1, or RET genes, and no amplification of the MET gene.

**Fig. 3:**
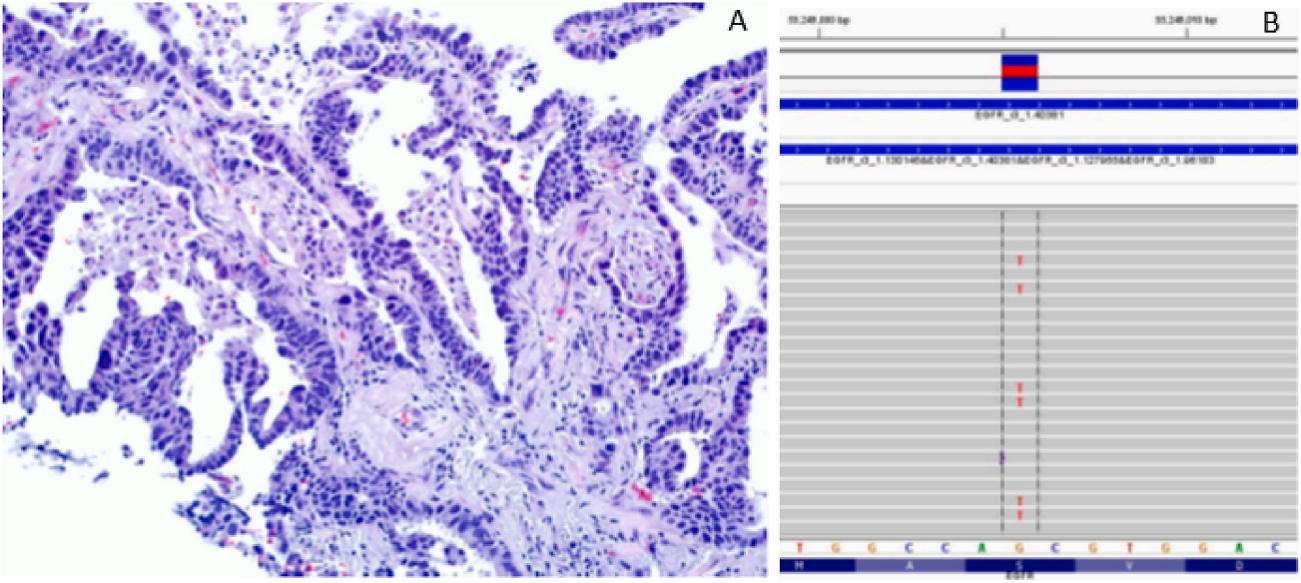
**A-B**: Higher power image of lung needle core biopsy showing well-differentiated adenocarcinoma **(A)** with the corresponding targeted NGS revealing two sensitizing EGFR mutations including S768I **(B)** & G719C (not shown).

**Fig. 4:**
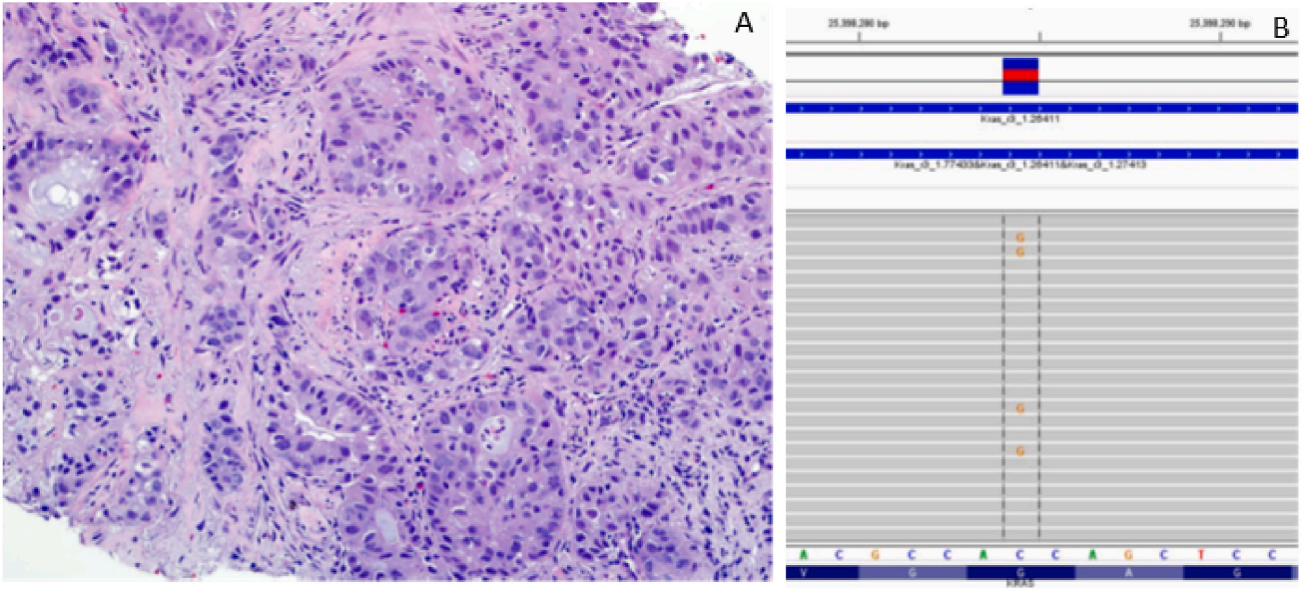
**A-B**: Higher power image of lung needle core biopsy showing moderately-differentiated adenocarcinoma **(A)** with corresponding targeted NGS revealing a c.35G > C (p.G12A) mutation in codon 2 of KRAS **(B)**.

Because of the unique morphologic and molecular profiles in this heterogenous tumor that raised the possibility of a collision tumor, and after presenting the case at a multi-disciplinary tumor board session, it was decided to separately macro-dissect the well-differentiated and the moderately-differentiated areas of tumor and repeat the targeted NGS panel on separately extracted DNA samples representing well and moderately differentiated components of tumor. The repeat NGS results identified the two sensitizing EGFR mutations including G719C and S768I in the well-differentiated, TTF1-positive portion of tumor (Fig. 3A and B), and the KRAS G12A in the less differentiated component of tumor (Fig. 4A–B).

The NGS findings supported the two separate primaries in this heterogenous appearing needle biopsy. The immunohistochemical and molecular findings are summarized in Table 1.

**Table 1:** Summary of key immunohistochemical and molecular findings of the collision lung tumor.

	IHC	Molecular Profile (NGS)
Well-differentiated component	TTF-1/Napsin-A +, CK7 +, small subset CK20 +, SATB2 focally +, CDX2 -, PAX8 -, GATA3 -	Two sensitizing EGFR
		mutations (G719C and S768I variants)
Moderately-differentiated component	CK7 +, small subset CK20 +, SATB2 focally +, TTF-1/Napsin-A -, CDX2 -, PAX8 -, GATA3 -	KRAS G12A mutation

The patient deteriorated rapidly during the inpatient workup secondary to disease progression and was transitioned to hospice care without further cancer-related medical or surgical intervention.

## 3 Discussion

Lung cancer is the leading cause of cancer death in the United States and world, and NSCLC accounts for majority of cases [1,2]. Lung adenocarcinoma tends to be heterogeneous by histomorphology as well as immunophenotypically. As previously discussed, tumors with or without multiple primaries present as separate tumor nodules, but with intermittent cases presenting as contiguous/collision tumors. Distinguishing collision tumors from tumor heterogeneity can present challenges on morphologic and immunophenotypic grounds alone, and molecular profiling can serve as a useful tool with this differential diagnosis. Furthermore, testing for targetable driver mutations in lung adenocarcinoma including epidermal growth factor receptor (EGFR) and KRAS mutations is standard of care because of the critical diagnostic and therapeutic implications [4,5].

While EGFR and KRAS mutations have been generally considered mutually exclusive driver mutations, there have been rare isolated case reports of lung adenocarcinomas harboring concurrent mutations in these apparently mutually exclusive genes [1,[4], [5], [6], [7], [8]]. EGFR and KRAS mutations are among the major actionable genetic alterations in lung adenocarcinomas. College of American Pathologists, the International Association for the Study of Lung Cancer, and the Association for Molecular Pathology recommend molecular testing for EGFR and KRAS mutations, among others [5]. These mutations often show certain clinicopathologic features and may be associated with specific molecular abnormalities. EGFR mutation is more commonly seen in adenocarcinomas, with papillary, micropapillary, acinar, and lepidic patterns, and in women who have no history of tobacco use or light smokers, while the KRAS mutation is often observed in adenocarcinomas with solid pattern, mucinous adenocarcinoma, and in men with a history of tobacco use [3,5,9].

The most common activating EGFR mutations are exon 19 deletions and exon 21 L858R missense mutation. Together, they account for up to 90 % of cases and exhibit sensitivity to tyrosine kinase inhibitors (TKIs) [1,6,7]. Uncommon EGFR mutations include the sensitizing point mutations of exon 18 such as G719C, G719S, G719A in approximately 3 % of NSCLC, exon 21 L861Q, and exon 20 S768I. Interestingly, the S768I mutation has been found to coexist with the exon 18 G719C, G719S, or G719A mutations [1,6,7]. Although there is limited data in the optimal management in patients with uncommon mutations, current guidelines suggest they be approached similarly to the common EGFR mutations and given EGFR TKIs. Ricciuti et al. reported a case of long-term survival with erlotinib in NSCLC harboring the uncommon EGFR G719S and a concurrent KRAS G12C mutations [6]. Furthermore, Tanizaki et al. reported a case where afatinib was clinically effective for the treatment of NSCLC in a non-smoker with the uncommon EGFR G719C and S768I mutations with a concurrent KRAS mutation [7]. The most common KRAS mutations include G12C, G12V, G12D. KRAS mutations are found in approximately 15–30 % of NSCLC and are generally larger and poorly-differentiated with an overall worse prognosis. FDA has approved administration of targeted KRAS G12C inhibitors; however, no FDA-approved targeted therapies are available for other KRAS variants at this time. Because KRAS is a downstream mediator of EGFR signaling, KRAS mutated tumors confer resistance to EGFR TKIs [[5], [6], [7]].

The presence of two or more lung tumors raises the differential diagnosis that includes intrapulmonary metastases of one primary or synchronous or collision tumors, particularly when tumors exhibit different histomorphology and/or different immunophenotype. This often creates a diagnostic challenge, and separate targeted DNA sequencing can serve as a useful ancillary tool in this setting. Acosta and colleagues studied the role of NGS in composite tumors, defined as mixed clonal tumors with divergent phenotypes, collision of two independent tumors adjacent to each other, and tumor-to-tumor metastasis, with original diagnosis made by clinical information, histology, and immunophenotype and limited molecular studies in two of four cases [10]. Their study demonstrated that NGS has a role, along with histology and IHC, in the diagnostic workup of these rare and challenging composite tumors [10] Murphy et al. sequenced 41 tumor samples from patients with known independent primary tumors and metastatic tumor, and they found concordance between histology and genomic data occurred in the majority of samples; however, discrepant samples were resolved by NGS, exemplifying the utility of genome sequencing in differentiating primary tumors from metastatic disease in lung cancer [11]. Other groups have shown the benefit of genomic analysis, with both limited customized gene panel and comprehensive NGS panels, to assess and distinguish this challenging differential diagnosis [[12], [13], [14]].

To our best knowledge, this is the first case report of a patient initially thought to have concurrent EGFR and KRAS mutations in a heterogeneous lung adenocarcinoma, but repeat targeted NGS panel in the separately macro-dissected well-differentiated and moderately-differentiated components confirmed a collision tumor. The well-differentiated adenocarcinoma expressed TTF1, and NGS revealed two sensitizing EGFR mutations, including the uncommon G719C and S768I variants. The moderately-differentiated adenocarcinoma was negative for TTF1, and NGS identified KRAS G12A mutation. The patient presented with advanced, metastatic disease to the left acetabulum with histomorphologic features similar to the KRAS mutated, moderately-differentiated component of the lung biopsy, supporting metastasis from the well-documented more aggressive component of the collision tumor. Unfortunately, the patient rapidly declined and was transitioned to hospice care without any tumor-targeted therapy.

## 4 Conclusion

Distinction of collision tumors from a single tumor with heterogeneity can be difficult on morphologic grounds, and sequencing analysis of DNA extracted from the entirely sampled tumors may fail to separate collision tumors from tumor heterogeneity. We have shown in the current case that targeted molecular profiling on DNA obtained from separately micro- or macro-dissected foci of heterogenous appearing tumors can prove helpful in separating true tumor heterogeneity from synchronous/collision tumors and increases diagnostic accuracy and improves patient care.

## CRediT authorship contribution statement

**Mary M. Torrez:** Writing – original draft. **Khalil Sheibani:** Writing – review & editing. **Mohammad A. Vasef:** Conceptualization, Writing – review & editing.

## Declaration of competing interest

The authors declare that they have no known competing financial interests or personal relationships that could have appeared to influence the work reported in this paper.
